# Do Double-Edged Swords Cut Both Ways? The Role of Technology Innovation and Resource Consumption in Environmental Regulation and Economic Performance

**DOI:** 10.3390/ijerph182413152

**Published:** 2021-12-13

**Authors:** Qian Zhou, Meng Shi, Qi Huang, Tao Shi

**Affiliations:** 1 Economics School, Zhongnan University of Economics and Law, Nanhu Avenue 182, Wuhan 430073, China; Z0005072@zuel.edu.cn; 2 Department of International Cooperation, China National Academy of Governance, Dayouzhuang 100, Beijing 100091, China; shimeng0554@163.com; 3 Zhengzhou Central Sub-Branch of People’s Bank of China, Shangwu Road 20, Zhengzhou 450000, China; huangqixl@163.com; 4 Economics Institute, Henan Academy of Social Science, Fengchan Road 21, Zhengzhou 450002, China

**Keywords:** environmental regulation, economic performance, technology innovation effect, resource consumption effect, spatial instrumental variable panel models

## Abstract

The Guangdong–Hong Kong–Macao Greater Bay Area (GBA) is one of the significant regions with the strongest economic vitality in China. This study focuses on environmental regulation in the eleven Greater Bay Area cities to explore the relationship between it and economic performance for the period 2000–2016. In doing so, we employ spatial panel models (including the spatial instrumental variable method) to investigate the nonlinear relationship between economic growth and environmental regulation. We confirm the existence of a U-shaped relationship between economic growth and environmental regulation in the Greater Bay Area. In the first half of the inverted U shape, the higher the economic development level, the stronger the environmental regulation strength; however, the latter begins to decrease after the peak point. The doubled-edged sword does not cut both ways. This paper verifies that technology innovation and resource consumption are two important mechanisms. Further, we find that both economic growth and environmental regulation have negative spatial externalities; innovation has a positive impact on the environmental regulation of the local city as well as surrounding cities, while resource consumption is on the contrary. In conclusion, this paper provides policy recommendations to further promote economic growth and environmental technologies, and to enhance energy efficiency in GBA.

## 1. Introduction

Chinese economic development is turning from a high-speed stage to a high-quality stage with more emphasis on green development. The economic development has been stable and high-speed for about 40 years since 1978. In 2019, the GDP achieved 99.08 trillion CNY with a growth rate as high as 6.1 percent, which means the economic development in China has a good momentum. However, the quality of the economic development in China is not well, as the GDP per capita in China is far behind that of the developed countries such as U.S. and Japan, and the environmental problems induced by the economic development are still serious, especially in China’s developed regions. In 2019, the PM2.5 average concentration in the Beijing–Tianjin–Hebei Region, Yangtze River Delta, and Pearl River Delta was 50 ug/m^3^, 41 ug/m^3^, and 42 ug/m^3^, respectively, which is much higher than the WHO standard of 10 ug/m^3^. At the same time, the negative externalities caused by environmental problems, such as economic loss and brain drain [1,2,3], have become more obvious [4], and the environmental problem caused by the extensive development model has come back to affect economic development step by step. Actually, according to the calculation by Wu, S. and Han, H. [5], the green GDP of China in 2019 was about 27.04 trillion CNY, about 27.29% of the total GDP calculated by the National Bureau of Statistics in the same period, which shows that the negative externality of the environment problem has an increasingly binding effect on economic development.

The environment, as quasi-public goods, is one of the key areas for the government’s public governance, and the intensity and direction of government environment policy affect the result of regional environment governance. Therefore, most scholars consider the government policy to be the primary factor in the environmental governance problem [6]. In terms of the economic development process of countries around the world, environment regulation can constrain economic development, which is why the government hesitates in environmental regulation. Environmental regulation and economic development have a negative correlation, shown as an economic development model of treatment after pollution [7,8]. Furthermore, that model evaluates the “environmental treatment is equal to economic development” model for China’s high-quality development stage as well as in most developed countries, which shows the positive relationship between the environmental regulation and economic development. Actually, current research shows that the relationship between environmental regulation and economic development is an inverted U shape [9], and there also exists a significant threshold effect [10]. Furthermore, regardless of any type of environmental regulation and economic development, environmental regulation has to be an important factor effecting economic development [11,12].

However, there is little research analyzing the bidirectional relationship between environmental regulation and economic development in China, especially research that tests their endogenous relationship by spatial instrumental variable method, and explores the mechanism between them. Based on the view above, this article analyzes the relationship between environmental regulation and economic development in Guangdong–Hong Kong–Macao Greater Bay Area from 2000 to 2016. The research above can distinguish the importance of different variables properly, and identify the reverse causality relationship between environmental regulation and economic development. This new dataset allows the following contributions to be made. (1) The paper explores the potential relationship between environmental regulation and economic development in sample regions, by using normal panel model and spatial panel model. (2) In order to solve the endogenous problem of reverse causality, we use the instrumental variable method to expect the maximum reliability of the result, and the result shows that there is a relatively stable relationship between environmental regulation and economic development in an inverted U-shape pattern. (3) The paper examines the mediating effect of technological innovation and resource consumption during the interaction between environmental regulation and economic development.

The remaining part of the paper is structured as follows. Section 1.1 provides a brief literature review on urban environmental regulation and its measurement. Section 2 describes the data used in the paper, formalizes the empirical framework, and presents the spatial panel data model. In Section 3, we report the empirical estimation results. Based on different regression analyses, we study the mechanisms in Section 4, and then summarize the paper in Section 5.

### 1.1. Environmental Regulation and Economic Performance: A Brief Overview

#### 1.1.1. The Background of Environmental Regulation

The term “environmental regulation” originates as a law of human activities with a view to preventing them from damaging the natural environment, and the main difficulties to cope with are uncertainty and economic loss [13,14].

In order to solve these difficulties, environmental regulation has generally been treated as an administrative order or regulatory instruments, such as industry monitoring and regulating, license, permission quality objective with environmental requirement, emission limits, and orderly control of energy. In addition, there exists another environmental regulation instrument, called an economic instrument, such as demonstrated in pollution rights and charges [15,16], and the German ‘ecotax’ on energy consumption can serve as an example. However, economists criticize the regulatory instrument as being inefficient [17,18], while the economic instrument is more efficient, for it is up to the entrepreneurs to pay and invest or not.

In addition to formal or informal environmental regulations, the market actors, such as enterprises or individuals, may protect the environment autonomously through self-control, such as through a market-oriented mechanism in environmental protection [19,20].

#### 1.1.2. Environmental Regulation and Economic Growth

With rapid economic growth, productive resources such as oil and nature gas have reduced sharply, while CO2 emissions have increased. Therefore, the contradiction between economic growth and environmental pollution is increasingly prominent, which is shown in the environmental EKC curve, i.e., after the top point of economic growth, environmental pollution is aggravated accordingly [21]. It is precisely the nonlinear relationship between environmental pollution and economic growth that has attracted the attention of many scholars to environmental regulation in the process of economic growth, to pursue the balance between environmental regulation and economic growth.

However, the relationship between environmental regulation and economic growth is unbalanced in most cases. Some scholars consider that environmental regulation goes against economic growth. The stricter the environmental regulation, the more that enterprises will need to invest in clean technology and green energy, and the percentage of punitive tax of environment pollution will obviously increase in the production process. The punitive tax will directly increase the production cost, and some enterprise will stop production or go bankrupt, which leads to unemployment and other social problems [19]. Meanwhile, some scholars believe that environmental regulation is conducive to economic growth. Environmental regulation has a reversed transmission effect that will force enterprises to improve production technology and adopt new cleaner technology to satisfy the higher environmental regulation, and it can spin off into the amount of clean technology and green energy industry, which means to use green technology to promote economic growth [22,23,24]. Moreover, environmental regulation has a direct effect on the competition and efficiency of the enterprise and industry development, and there exists a Porter Hypothesis [25]. For example, the data of 15 Korean industries from 1983 to 1993 show a clear negative effect between market power and the environmental regulations’ contribution to the growth of production efficiency in South Korean [26]. However, environmental regulation has an obvious positive effect on green production industry, but has a lag effect on heavy-polluting industry, there is an inverted U curve correlation between environmental regulation and production efficiency, and there exists a three-thresholds effect [27]. In most cases, the relationship between environmental regulation and economic growth is more like an inverted U curve or environmental EKC curve [28].

However, the evidence from OECD demonstrates that the EKC hypothesis is not established, which means economic growth cannot solve the environmental degradation problem [29], and there may exist an inverse causality relationship between economic growth and environmental regulation, although few empirical articles support this result.

#### 1.1.3. Environmental Regulation, Technological Innovation, and Economic Growth

There is a big debate between environmental regulation and technological innovation. Some scholars believe that environmental regulation will not be conducive to technological innovation as it potentially increases the production cost, while others believe that environmental regulation can be conducive to technological innovation by reducing regulatory risk [30,31,32]. However, more research shows that obviously there exists an inverted U-shaped curve between environmental regulation and technological innovation, i.e., environmental regulation has an offsetting effect on technological R&D and innovation capabilities in the short term, and has a compensation effect in the long run by promoting enterprises to improve the technological innovation capabilities to reduce the environmental control cost under the pressure of stricter environmental regulation [33,34]. In the earlier stage, the strength level of environmental regulation will be higher to abate environmental pollution, while the abatement cost of enterprise increases obviously, and under taxes and standards, only the current least-cost technology is used and developed, implying a lock-in into a possibly inferior technology [35,36]. Especially, stricter environmental regulatory frameworks in emerging economies are not only combating pollution, but also shifting the innovation activities of manufacturing firms towards building a stock of knowledge in environmental protection, and generating disruptive eco-innovations [37]. In the long run, the core function of green technological innovation can be established in the ecological modernization by using environmental regulation [38], and enterprises will be pushed to bring technological innovation up to the higher environmental regulation standards (as ISO14000) [39], eventually protecting the environment precisely [40].

At the same time, technological innovation is an important factor to accelerate the economic growth according to the theory of classical economics, especially to strength the innovation preference of government and to increase the economic growth rate obviously [41]. In the short run, there exists a strong causal link between technological innovation and economic growth, which are not always synchronous; in the long run, innovation stimulates economic growth [42], and information communication technology as well as financial development are positive driving factors to economic growth [43]. In addition, the effect of technological innovation on economic growth varies greatly among regions. Technological innovation contributes simultaneously to sustainable economic development, social advance, and the environmental condition only in the case of rich countries; however, it only affects the economic and environmental dimensions in the middle-income countries, and no impact is found in the case of low-income countries [44].

Furthermore, technological innovation is an important mechanism for environmental regulation to influence economic growth [45]. Under the impact of innovative compensation, compliance cost, and energy rebound effects, when green technological innovation (GTI) efficiently improves the eco-efficiency (EE), inappropriate environmental regulation will weaken the marginal benefits of GTI. When an “energy rebound effect” occurs, moderate environmental regulation will be found to assist in reducing the harmful influence of GTI [46]. Therefore, under the environmental regulation, technological innovation can promote economic growth.

#### 1.1.4. Environmental Regulation, Resource Consumption, and Economic Growth

Oil, coal, and other energy resources are important factors for economic growth, and many studies prove that the strengthened environmental regulation is a key factor in reducing environmental pollution caused by resource consumption [47], and in promoting green economic growth. In order to reduce the potential environmental pollution caused by excessive use of nature resources, and to meet the governmental control standard of environmental pollution for the producing and living conditions, it is imperative for energy-consuming enterprises to improve the efficiency of energy usage, and reduce the emission of CO2, PM2.5, and other polluting gas [48]. On the other hand, it is important to increase the R&D and investment of clean energy and green technology, such as hydrogen, solar energy, wind energy, and tidal energy, to strengthen the replacement of traditional fossil energy with clean energy [49].

However, there is also a big debate between resource consumption and economic growth. Some scholars believe that there exists an obvious bidirectional Granger causality relationship between resource consumption and economic growth. Especially in the developing countries, the bidirectional causality relationship between coal consumption and economic growth is more obvious [50,51], and there exists the feedback hypothesis between biomass consumption and economic growth [52,53]. Some scholars believe that there is a unidirectional causality effect between resource consumption and economic growth, and resource consumption is conducive to the economic growth [54,55]. However, energy conservation policies do not have an adverse effect on economic growth in both the short and intermediate run, while their effects are negative in the long run [56].

#### 1.1.5. Summary

Although many studies elaborate on the relationship between environmental regulation and economic growth, they ignore the analysis of intermediate channels. In this study, we aim to determine whether technological innovation and resource consumption can bridge environmental regulation and economic growth, and identify the policy changes needed for their economic integration. The main contributions are in (1) using the data of 11 cities in Guangdong–Hong Kong–Macao Greater Bay Area from 2000 to 2016 to analyze the relationship between the environmental regulation and economic development; (2) identifying the two intermediary mechanisms involved using spatial analysis; (3) ensuring the accuracy of the regressions involving instrumental variables to solve the endogenous problem; (4) providing some interesting conclusions and policy recommendations.

## 2. Materials and Methods

### 2.1. Research Methods

Based on the research about environmental regulation and economic development [57], combined with the purpose of this article, we established an environmental regulation model as follows:

lnregu_it_ = f(lnpgdp_it_, lnpgdpsq_it_, other variables_it_) + v_it_ (1)

In model (1), the subscript it stands for city i in year t, respectively. regu refers to the environmental regulation, and lnregu is the logarithm value of regu accordingly, and the same below; pgdp refers to the economic development, and pgdpsq refers to the square of the pgdp, as pgdpsq = pgdp^2^; v_it_ refer to the error item; other variables are the control and instrumental variable.

Based on model (1), this article tries to test whether there is a nonlinear relationship between environmental regulation and economic development, which is different from the result of the previous research. It is the most important content in this article.

As we know, when the economic development in a good condition, it may have two impacts on the environment: the local government may attract investment, promote employment, and stimulate development at the expense of environment, or it may strengthen the environmental protection and improvement through the good economic condition, which is the economic operational foundation leading to the nonlinear relationship between environmental regulation and economic development.

Furthermore, we realize that not only will economic development affect environmental regulation, but also environmental regulation will become the important indicator that constrains the economic development potential, which is supported by most current literature. Therefore, there exists a reverse causality relationship between economic development and environmental regulation in theory. In order to solve the problem above, this article explores the relationship between the economic development and environmental regulation by using the traditional panel model and spatial panel model at first. Then, for the endogenous problem of reverse causality, we adopt the instrumental variable method in order to maximize the reliability of the results. Testing the relationship between economic development and environmental regulation is the important innovation of this article.

In the model (1), the control variables are the population size, population density, industry structure, the number of the college students, public health resource, and the condition of construction industry, and so on.

### 2.2. Variable Selection

#### 2.2.1. Dependent Variables

Environmental regulation (regu). We chose industrial solid waste generation, industrial waste gas discharge, total waste water discharge, and household garbage clearance and transportation volume as four basic indicators [58], and then weighted these variables by using improved entropy method, to calculate a standardized environmental regulation index.

#### 2.2.2. Core independent Variables

The economic development level and its squared term. We added the economic development level and its squared item to analyze the nonlinear relationship between environmental regulation and economic development, which is the important innovation in this article [59]. The specific form is the logarithm of GDP per capita.

#### 2.2.3. Control Variables

(1) Population size (pop). Population size directly affects the environmental regulation, and reflects the vitality of the economic development. The more population and vitality, the more closed the relationship between population and environmental condition.
(2) Population density (density). For the different city areas, population density can directly reflect the environmental regulation from a cluster view, which is different from population size.
(3) Industry structure. We chose the variables of the percentage of second industry in GDP and the percentage of tertiary industry in GDP [60] to analyze in which direction and to which degree those two industries affect environmental regulation.
(4) The number of college students (stu). This indicator is used to appraise the educational conditions in one city. We believe that the urban citizens with good educational conditions will be conducive to the development of environmental regulation.
(5) Public health resource. It includes three indexes: the number of health agencies, beds, and personnel. We believe that the city with better public health resources can affect environmental regulation by two ways: in one aspect, the more medical waste, the more pollution, which is not conducive to the development of environmental regulation. In the other aspect, the better public health resource, the better economic development in one city, which will require more environmental protection in favor of environmental regulation [61].
(6) The condition of construction industry. The construction industry can reflect the infrastructure level of one city, and this indicator measures the development status of the environmental regulation: The more the development in construction industry, the more the development in environment, which will affect the environmental regulation.

#### 2.2.4. Intermediate Mechanism Variable

(1) Technology innovation. The better the development in economy, the higher the demand and requirement for technology, which will promote the development of innovation and then change the carrying capacity of the environment.
(2) Resource consumption. In general, the initial economy development is more likely to be the result of the increasing factor input. As the economy is developing, the dependence on resource gradually decreases, and this process will have an impact on the environmental regulation [62].

#### 2.2.5. Instrument Variable

Since the endogenous problem will affect the analyses of the relationship between the economic development and environmental regulation, this article introduced the instrument variable to avoid endogenous variables. It is noticed that many landmark major projects have been constructed in Guangdong–Hong Kong–Macao regions to promote the economic development during the sample observation period from 2000 to 2016, especially the construction of the high-speed railway and the Hong Kong–Zhuhai–Macao Bridge. This phenomenon is highly correlated with the economic development, but not with the environmental regulation, so it meets the requirements of exogenous variables. We introduced the instrument variable based on the above, and the specific approach is specified as follows: we used the actual number of the high-speed railways as source of the instrument variable for 9 inland cities, and the construction of the Hong Kong–Zhuhai–Macao Bridge as the source of the instrument variable for Hong Kong and Macao.

### 2.3. Data Sources

Considering the data availability, this article used the panel data of 11 cities in Guangdong–Hong Kong–Macao Greater Bay Area from 2000 to 2016 to analyze the relationship between the environmental regulation and economic development. The data originates from City Statistics Yearbook and information collected by crawler technology. In this article, interpolation method was used to supplement missing value, and the variables related to price were deflated by using the base period of 2000. Table 1 shows the descriptive statistics of related variables.

### 2.4. The Relationship Pattern between the Environmental Regulation and the Economic Development

**Figure 1:**
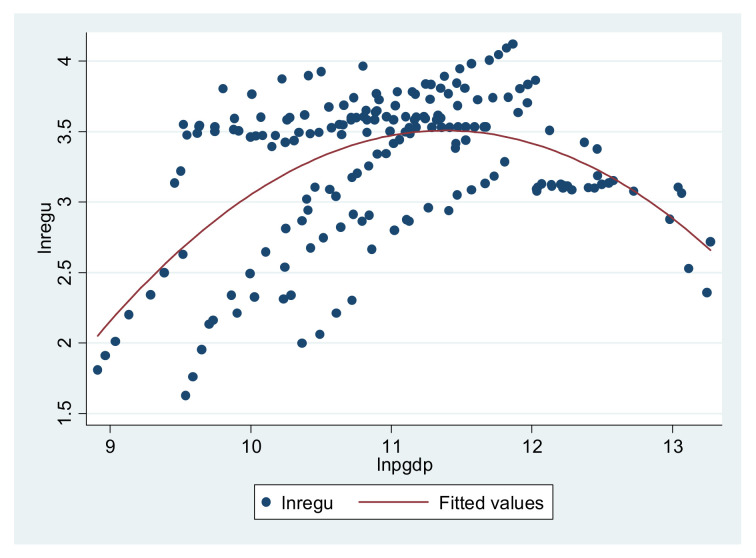
The relationship pattern between environmental regulation and economic development.

Before empirically examining the basic curve relationship between the environmental regulation and economic development, we observed whether the basic relationship between the two meets the conception of this article or not by using a scatterplot. This is important to the model construction for this article. Figure 1 shows the relationship between the environmental regulation and economic development. The horizontal axis is economic development, and the vertical axis is environmental regulations. We found that the nonlinear inverted U-shaped curve relationship is obvious, which provides a strong support for the following analysis.

### 2.5. Econometric Model and Spatial Concerns

In order to construct an integrated economic circle, the Guangdong–Hong Kong–Macao Greater Bay Area (short for Bay Area) is based on the short distance among cities in the Bay Area, in order to promote urban agglomeration and regional economic development and to enhance the connection among Hong Kong, Macao, and inland cities in China. Hence, the mission of the Bay Area is extremely significant and prominent in the development process of China. Artificially splitting the connection between the cities in Bay Area may lead to biased analysis result. Considering that the normal econometric method cannot efficiently capture the characteristic, this article adopted a spatial econometric model to observe the necessity of integrated development in the Bay Area, the correlation and spillover effect of environmental regulation. Furthermore, we introduced instrument variables to solve the endogenous problem caused by the reverse causality.

Before using the spatial econometric model, we constructed a spatial matrix. The spatial matrixes used in this article includes geographical matrix (W1), economic matrix (W2), and geo-economic matrix (W3). The reasons are as follows: (1) the environmental regulation among cities will be influenced by nearby cities, and it is necessary to consider the geographical distance. (2) The environmental regulation in a city is influenced not only by itself but also by the economy of nearby cities, and its economic distance should be considered. The higher the economic closeness rate, the shorter the economic distance. (3) In addition to the two impacts above, this article considered the co-influence of economy and geography, and introduced the geo-economic matrix.

Compared to the linear model [63], the nonlinear model can better show the nonlinear relationship between different variables, especially the inverted or normal “U” type of EKC (Environmental Kuznets Curve). The traditional model cannot reflect the spatial relationship between regions, and only shows the relationship between different variables [64,65], while the environmental issue shows more in inner-regions. The advantage of the spatial model is that it shows the regional relationship between variables, especially the spatial spillover effect and decomposition. In the model choice, this article used the spatial autoregressive model (SAR), spatial error model (SEM), spatial Dubin model (SDM), and spatial IV model (SIV) to observe the following aspects: the interaction of environmental regulation among cities; except the control variable, the spillover effect among different cities from the other variables that influence the environmental regulation. The environmental regulation correlates not only with economic development of the city itself, but also with nearby cities. Based on the above, this article used spatial IV model, and solved the endogenous problem by using the proxy variable of economic development.

Based on the Equation (1) and variable-choosing process above, referring to Chen, J. and Zhou, Q. [66], and Elhorst, J. P. et al. [67], we set the spatial econometric model as follows:

(2) $$\begin{array}{l} \ln regu_{it}=\tau \ln regu_{it-1}+\delta W_{N}\ln regu_{it}+\eta W_{N}\ln regu_{it-1}\\ +\alpha_{1}\ln pgdp_{it}+\alpha_{2}\ln pgdpsq_{it}+\alpha_{3}\ln pop_{it}+\alpha_{4}\ln manu_{it}+\\ \alpha_{5}\ln sevi_{it}+\alpha_{6}\ln dens_{it}+\alpha_{7}\ln stu_{it}+\alpha_{8}\ln health_{it}+\\ \alpha_{9}\ln bed_{it}+\alpha_{10}\ln doctor_{it}+\alpha_{11}\ln consp_{it}+\nu_{it} \end{array}$$

(3) $$\mu =\rho W_{N}v_{T}+\xi_{t}$$

In Equations (2) and (3), τ is the space delay factor for dependent variables capturing the spillover effects from neighboring cities, t represents the time length, N is the number of cities, and W_N_ is the N-order spatial weight matrix. μ is the space error term, ρ is the spatial error coefficient, v is the vector dataset containing the independent and identically distributed error. Furthermore, τ, δ, η represent the time lag factor, the space lag factor, and the time–space lag factor, respectively.

## 3. Results

### 3.1. The Economic Development Interaction

Firstly, we analyzed the interaction relationship of economic development among cities in the Bay Area, and the columns (1)–(3) in Table 2 show the results. The result shows that whether it is a geographically adjacent city or an economically adjacent city, the coefficient of local city is significantly negative in terms of economic development. It indicates that there is an obviously competitive relationship between geographically adjacent cities and economically adjacent cities. The economic development interaction among cities is far from a win–win situation, and only the cities with geographical and economical adjacency will have a positive influence on the development of the local city.

Objectively, the geographical segregation by the administrative division cannot be changed towards better. However, from the regional integration view, the cities in the Bay Area are closed to each other, which means there is a large room for improvement. In contrast, we should pay more attention to economic separation, and the economic growth model of the beggar-thy-neighbor is against integration and the original intention of the development in Guangdong–Hong Kong–Macao. Therefore, the governments in the Bay Area need to make more effort on coordination and cooperation.

### 3.2. The Environmental Regulation Interaction

The interaction of the economic development among cities in the Bay Area is subjective, while the interaction of the environmental regulation is more objective, and is an unchangeable fact in an ecological system. The columns (4)–(6) in Table 2 show the results of the interaction relationship of environmental regulation. In the result, whatever the environmental regulation is for the geographical nearby city or the economic nearby city, the coefficients of the local city is significantly negative, and if these two are nearby simultaneously, the coefficient is not significant. From the environmental view, the results above mean that the competitive relationship between the cities in Bay Area is still strong, and this is understandable: under certain conditions, the objective environmental regulations of the environment are limited, and the beggar-thy-neighbor model may be limited by the existing environmental constrains. Nevertheless, it is necessarily to reverse this situation to promote the regional development.

### 3.3. The Relationship Analysis between Economic Development and Environmental Regulation: Baseline Results

Table 3 shows the normal panel data estimation results of the influence of economic development on environmental regulation, among which columns (7)–(8) show the results of OSL, and columns (9)–(10) are the results of fixed-effect regression.

From Table 3, we can see that the impact of economic development on environmental regulation presents an inverted U shape, and it rises first and then decreases, which is consistent with the curve in Figure 1. In the first half of the inverted U shape, the higher the economic development level, the stronger the environmental regulation strength, and the latter begins to decrease after the peak point. The result relates to the definition and calculation method of the environmental regulation, and in this article, the economic development is accompanied by the industrial development process. In the initial improvement stage, there is a phenomenon of sacrificing the environment to promote economic development, and during this period there is more and more industrial solid waste, waste gas emission, water discharge, and living garbage. The environmental regulation based on these above four indicators will be stronger, and may subsequently be abated due to the industrial structure adjustments and technological improvement, which leads to the U-shaped environmental regulation.

### 3.4. The Estimation Result and Analysis of Spatial Panel Regression

As mentioned above, the study is based on the spatial econometric model, and uses the general regression results in Section 3.3 as the benchmark reference for the spatial regression results. Table 4 is the spatial regression result of economic development and environmental regulation, using a spatial geographical matrix in columns (11)–(14), a spatial economic matrix in columns (15)–(18), and a spatial geo-economic matrix in columns (19)–(22). The result shows that the inverted U-shaped relationship between the economic development and environmental regulation in the spatial model still exists. We consider the SAR model, SEM model, SDM model, and spatial IV model, but focus on the analysis of the spatial IV model.

Firstly, by using the instrument variable, the regression coefficient of economic development becomes smaller than before, and may efficiently solve the problem of overestimating the regression result of the correlation solve effectively. From the spatial correlation, we find the negative externality in economic growth. The reason lies in that there are radiation effects and siphon effects among cities. At present, the reason why economic growth still shows a negative spatial externality is that the siphon effect exceeds the radiation effect. This is consistent with the basic facts of China. The core cities of urban agglomerations often form a certain degree of siphonic effect on surrounding cities due to their own political and geographic advantages.

Secondly, comparing with the spatial correlation, we find that after using the instrument variable, the results of these three matrix forms are still significantly negative. We believe that the construction of the high-speed rail and Hong Kong–Zhuhai–Macao Bridge plays an important role in promoting the integration of Guangdong, Hong Kong, and Macao, but when the environmental regulations of nearby cities are strengthened, its spillover effect will lead to the abatement of local environmental regulation. The environmental regulations of the Bay Area cities are still competitive with a beggar-thy-neighbor feature. In terms of promoting economic integration and environmental harmony in the Bay Area, the challenges are still obvious and prominent.

So far, this article has verified the curve relationship between economic development and environmental regulation as an inverted U-shaped pattern. In the next section, we further analyze the mechanism of economic development on environmental regulation.

## 4. Mechanism Analysis

In this article we believe that the possible paths by which economic development affects the environmental regulation are as follows: (1) Technological innovation. The more developed the economic is, the higher the requirement is for technology, thereby promoting the development of innovation, which will change the carrying capacity of the environment. (2) Resource consumption. Generally, the initial economy development is more likely to be the result of the increased factor input. With economic development, the dependence on resources will gradually decrease, and this process will have an impact on the environmental regulation. In order to verify these two guesses, this article explores the mechanism as follows.

### 4.1. Technological Innovation Effects

First of all, based on the definition of technological innovation effect, we observed the pattern of the scatter plot to construct the spatial panel model and then to verify the technological innovation effect.

**Figure 2:**
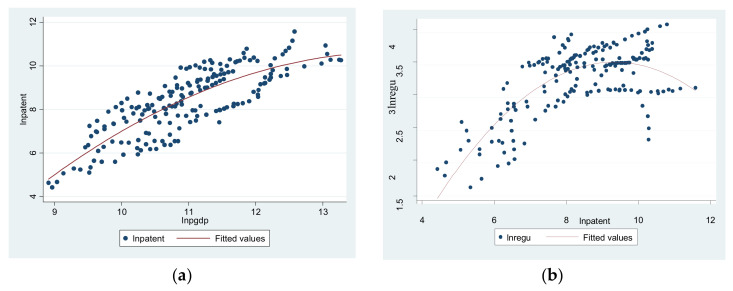
(**a**) The relationship between economic development and innovation. (**b**) The relationship between innovation and environmental regulation.

Figure 2a. shows that economic development promotes the innovation. The horizontal axis is economic development, and the vertical axis is innovation. In Figure 2b., the horizontal axis is innovation, and the vertical axis is environmental regulations. It shows that the impact from the innovation behavior on environmental regulation describes an inverted U-shaped tendency, which is demonstrated in the previous section.

According to Figure 2, we obtained the spatial panel model of technological innovation as

(4) $$\begin{array}{ll} \ln patent_{it} & =\tau \ln patent_{it-1}+\delta W_{N}\ln patent_{it}\\ & +\eta W_{N}\ln patent_{it-1}+\beta_{1}\ln pgdp_{it}+control_{it}+\nu_{t} \end{array}$$

In Equation (4), patent measures technological innovation, and others variables are the same as in Equation (2).

(5) $$\begin{array}{ll} \ln regu_{it}= & \tau \ln regu_{it-1}+\delta W_{N}\ln regu_{it}+\eta W_{N}\ln regu_{it-1}+\\ & \gamma_{1}\ln patent_{it}+\gamma_{2}\ln patentsq_{it}+control_{it}+\nu_{t} \end{array}$$

The corresponding spatial model of Equations (4) and (5) is the same as in Equation (3), which is omitted here to save space.

Table 5 shows the spatial regression result of the technological innovation effect. We can see that the Equations (4) and (5) have passed the test in different spatial matrix forms: economic development will promote innovation development, and the latter will cause an inverted U-shaped curve structure of environmental regulation. In terms of the spatial spillover effect, the innovation in nearby cities can enhance the innovation of nearby cities and will bring significant positive externalities. In contrast, the environmental regulation shows a competitive relationship among nearby cities. Hence, promoting innovation and enhancing the city’s environmental regulation can not only tap into innovation potential, but can also contribute to solve the environmental problem.

### 4.2. Resource Consumption Effects

**Figure 3:**
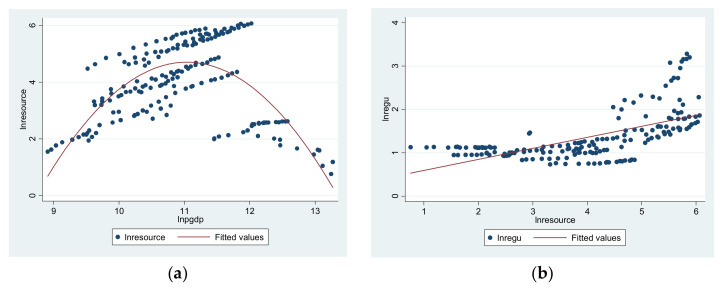
(**a**) The relationship between the economic development and resource consumption. (**b**) The relationship between the resource consumption and environmental regulation.

Figure 3a,b shows the scatter plot of the resource consumption effect. In Figure 3a, the horizontal axis is economic development, and the vertical axis is resource consumption. In Figure 3b, the horizontal axis is resource consumption, and the vertical axis is environmental regulations. In Figure 3a, we can see that as the economy develops, the resource consumption increases first and then gradually decreases, and economic development and resource consumption suggest an inverted U-shaped relationship. In Figure 3b, we can see that as the resource consumption increases, the environmental regulation has a tendency of increasing. Therefore, through Figure 3a,b, there is a U-shaped relationship between resource consumption and environmental regulation. It is not only consistent with Figure 1, but also proves that the resource consumption effect exists.

Based on the Figure 3a,b, we established a simple model as follows:

(6) $$\begin{array}{ll} \ln resource_{it} & =\tau \ln resource_{it-1}+\delta W_{N}\ln resource_{it}+\eta W_{N}\ln resource_{it-1}\\ & +\chi_{1}\ln pgdp_{it}+\chi_{2}\ln pgdpsq_{it}+control_{it}+\nu_{t} \end{array}$$

(7) $$\begin{array}{ll} \ln regu_{it} & =\tau \ln regu_{it-1}+\delta W_{N}\ln regu_{it}+\eta W_{N}\ln regu_{it-1}\\ & +\omega_{1}\ln resource_{it}+control_{it}+\nu_{t} \end{array}$$

In Equations (6) and (7), resource measures the resource consumption. The corresponding spatial model is the same as in Equation (3) and is omitted here to save space.

Table 6 shows the spatial regression result of resource consumption effect. W1, W2, and W3 refer to the geographic spatial matrix, economic spatial matrix, and geo-economic spatial matrix, respectively. Economic development and resource consumption show an inverted U-shaped tendency under the three circumstances, and resource consumption promotes the carrying capacity of the environment. This result is consisted with Figure 3, and also verifies the second mechanism in this article. Through the spatial spillover effect, we can see that the environmental regulation in nearby cities also shows an obviously competitive relationship of beggar-thy-neighbor. The geographically adjacent cities can compete for resource in the economic development process, while the economically adjacent cities will share the resource. In addition, economic development will always be at the cost of resource consumption to different extents, which is consistent with intuition.

### 4.3. Summary: Effect Decomposition

This article analysis the new curve relationship between economic development and environmental regulation, and verifies the impact mechanism of the technological innovation effect and the resource consumption effect. Here, this article further tests whether these two effects really play a role in affecting the mechanism and its impact degree. In order to obtain the direct effect and the indirect effect, we decomposed the total effect as Equation (8):

(8) $$\begin{array}{ll} \ln regu_{it} & =\tau \ln regu_{it-1}+\delta W_{N}\ln regu_{it}+\eta W_{N}\ln regu_{it-1}+\sigma_{1}\ln pgdp_{it}\\ & +\sigma_{2}\ln pgdpsq_{it}+\sigma_{3}\ln patent_{it}+\sigma_{4}\ln patentsq_{it}\\ & +\sigma_{5}\ln resource_{it}+\pi control_{it}+c_{i}+y_{t}+\varepsilon_{it} \end{array}$$

In formula (8), σ_1_ + σ_2_ measures the direct effect, and the indirect effect is calculated as

(9) $$Indirect\;effect=(\sigma_{3}+\sigma_{4})*innovation\;effect+\sigma_{5}*resource\;effect$$

Table 7 shows a comprehensive analysis of the technological innovation effect and the resource consumption effect. In the geographical matrix, the invert U-shaped curve between the economic development and environmental regulation also exists, and the coefficients of the first and second term become smaller (2.273 < 3.763, 0.084 < 0.174). In addition, the inverted U-shaped pattern of innovational technology and the positive impact of resource consumption also exist. This result shows that (1) the direct effect is 2.189; (2) the technological innovation effect and resource consumption effect are important mechanisms and reasons for economic development to affect the environmental regulation. In the economic matrix, the inverted U-shaped curve between the economic development and environmental regulation does not exist, while the inverted U-shaped curve between the technological innovation and environmental regulation holds. This result shows that (1) the direct effect is 0; (2) the technological innovation effect and the resource consumption effect are important mechanisms and reasons for economic development to affect the environmental regulation. The results above are the same when using the geo-economic matrix.

In the three spatial matrixes, Table 4 shows that the total effect is 3.589, 3.016, and 3.168, respectively. The technological innovation effect is 0.996, 1.071, and 1.063, respectively, as shown in Table 5, and the resource consumption effect is 0.299, 0.264, and 0.307, as in Table 6. According to Equation (9), we calculated the direct effect and the indirect effect, and all effect values are summarized in Table 8.

## 5. Conclusions and Policy Implication

### 5.1. Conclusions

The Guangdong–Hong Kong–Macao Greater Bay Area will not only be built into a vibrant world-class urban agglomeration and important support for the construction of the “Belt and Road” initiative, but will also be built into a high-quality living area suitable for living and working: a model of high-quality sustainable development in the blueprint of China. This paper used panel data of 11 cities of GBA from 2000 to 2016 to investigate the nonlinear relationship between environmental regulation and economic performance. Five main conclusions are presented:

(1) For economic performance, environmental regulation, and resource consumption, we find a competitive relationship among the geographically and economically neighboring GBA cities, which is harmful to regional integration. Compared with these, innovation has a significant positive spatial spillover effect.
(2) By using different econometric tools (especially spatial panel models), a U-shaped relationship between economic performance and environmental regulation has been found in GBA: with the development of per capita GDP, environmental regulation declines first and then rises after the bottom point.
(3) By using instrumental variables of number of high-speed railways in nine inland cities and construction of Hong Kong–Zhuhai–Macao Bridge in Hong Kong and Macao, the U-shaped curve for the economic–environment nexus among GBA cities has again been confirmed.
(4) Technological innovation and resource consumption are shown to be the important intermediate variables for the economic–environment nexus. On the one side, economic growth promotes innovation, and innovation brings the U-shaped curve of environmental regulation; on the other side, an inverted U-shaped relation exists between economic performance and resource consumption, while resource consumption negatively correlates with environmental regulation. These two mechanisms all illustrate the U-shaped relation between economic growth and environmental regulation in GBA.
(5) By decomposing the total effects, we find economic growth shows no direct effect on environmental regulation; technological innovation and resource consumption are confirmed to be important intermediate variables in the effect of economic growth on environmental regulation.

### 5.2. Policy Implication

On the one hand, we should further promote economic growth in GBA. According to the empirical results, a U-shaped relationship between the economic performance and environmental regulation does exist in GBA, implying that economic development has a positive impact on the environmental regulation in the long term, although the current growth of economy may make the environment deteriorate. In numerous studies exploring the nexus of economic growth and environmental pressures on the basis of EKC hypothesis or decoupling analysis [68,69], continuous growth of economy is always verified to be the essential promoting force of a better environment. The 13th Five-Year Plan (2016–2020) proposed the “New Normal” of coordinated development of economy and environment, with emphasis on environmental protection and resource efficiency while developing the economy. The development plan for Guangdong–Hong Kong–Macao Greater Bay Area released on 18 February 2019 also highlights the formation of a green and low-carbon production mode and lifestyle, aiming to provide a superior ecological environment for residents and promote the sustainable development of the Greater Bay Area.

On the other hand, we should also improve environmental technologies and enhance energy efficiency. The results in this paper show that technological innovation and resource consumption are essential mediating variables to link economic development and environmental regulation, and technological innovation has a significant positive spatial spillover effect, while economic performance, environmental regulation, and resource consumption have negative spillover effects. This creates an opportunity for us to explore the harmonious development of economic growth and environmental protection on the basis of innovation. In this light, local governments should adopt an innovation-driven strategy, establish a financial support system to develop low-carbon technologies and strengthen the construction of the technology innovation system. Moreover, upgrading the industrial structure, optimizing the energy structure, and establishing market-based carbon emission trading schemes are also effective tools to conserve the environment [70]. In response to these demands, the development plan proposes to construct an economic system driven and supported by innovation, and build “smart towns” characterized by low-carbon development. It also suggests to vigorously develop green energy, accelerate the use of natural gas and renewable energy, control the amount of coal consumption, and increase the proportion of clean energy. These regulations and measures would enhance energy efficiency and environmental-friendly technologies, promote the transformation of the economic development mode, and finally, strengthen the environmental regulation of the environment.

## Figures and Tables

**Table 1:** The descriptive statistics for the data of GBA during 2000–2016.

Variable	Description	Unit	Mean	SD	Min	Max
lnregu	Environmental regulation		3.266	0.528	1.628	4.119
lnpgdp	Economic development	CNY	10.98	0.928	8.912	13.27
lnpgdpsq	The square of the economic development		121.5	20.55	79.42	176.1
lnpop	Population size	ten thousand	6.817	2.978	3.777	15.98
lnmanu	The percentage of second industry	1	3.689	0.567	1.308	4.157
lnsevi	The percentage of tertiary industry	1	3.876	0.292	3.321	4.567
lndens	Population density	person/km^2^	7.363	1.250	5.425	9.971
lnstu	The number of college students	person	10.96	1.403	8.084	13.91
lnhealth	Health agencies	unit	6.438	1.094	4.094	8.244
lnbed	The number of beds in health agency	bed	9.328	0.964	7.002	11.30
lndoctor	The number of personnel in health agencies	person	9.578	0.709	7.667	10.77
lnconsp	The house price	yuan/sq.m	6.729	2.423	2.452	12.45
lnpatent	Patent grants	patent	8.356	1.508	4.419	11.58
lnpatentsq	The square of the patent grants		72.09	24.46	19.53	134.1
lnresource	Resource consumption		3.939	1.379	0.756	6.063

Note: The value variables are all deflated based on the year 2000 price. The sample size is 187.

**Table 2:** The interaction of economic development and environmental regulation.

Var.	(1)	(2)	(3)	(4)	(5)	(6)
	lnpgdp	lnpgdp	lnpgdp	lnregu	lnregu	lnregu
	W1	W2	W3	W1	W2	W3
wlnpgdp	−3.996 ***	−1.907 ***	14.513 ***			
	(0.521)	(0.529)	(3.144)			
wlnregu				−5.470 ***	−3.689 ***	12.039
				(0.554)	(1.066)	(7.385)
Control	Y	Y	Y	Y	Y	Y
Year	Y	Y	Y	Y	Y	Y
City	Y	Y	Y	Y	Y	Y
N	187	187	187	187	187	187
adj. R^2^	0.980	0.967	0.972	0.831	0.711	0.693

Note: Standard errors in parentheses. *** *p* < 0.01.

**Table 3:** The relationship between the economic development and environmental regulation (not the spatial result).

Var.	(7)	(8)	(9)	(10)
	lnregu	lnregu	lnregu	lnregu
	OLS	OLS	FE	FE
lnpgdp	4.167 ***	3.602 ***	3.021 ***	3.602 ***
	(0.755)	(0.814)	(0.706)	(0.814)
lnpgdpsq	−0.166 ***	−0.168 ***	−0.125 ***	−0.168 ***
	(0.035)	(0.039)	(0.032)	(0.039)
lnpop	0.111 ***	0.294	0.397	0.294
	(0.015)	(0.416)	(0.403)	(0.416)
lnmanu	0.113	0.200	0.353 ***	0.200
	(0.135)	(0.149)	(0.134)	(0.149)
lnsevi	1.009 ***	0.799 ***	1.126 ***	0.799 ***
	(0.270)	(0.300)	(0.282)	(0.300)
lndens	0.016	0.454 ***	0.378 ***	0.454 ***
	(0.045)	(0.130)	(0.131)	(0.130)
lnstu	−0.434 ***	−0.411 ***	−0.301 ***	−0.411 ***
	(0.049)	(0.060)	(0.059)	(0.060)
lnhealth	0.155 ***	−0.012	0.007	−0.012
	(0.029)	(0.047)	(0.044)	(0.047)
lnbed	−0.012	−0.064	0.349 **	−0.064
	(0.092)	(0.220)	(0.171)	(0.220)
lndoctor	−0.004	0.133	−0.134	0.133
	(0.110)	(0.174)	(0.159)	(0.174)
lnconsp	−0.034 *	−0.148 ***	−0.115 ***	−0.148 ***
	(0.018)	(0.025)	(0.026)	(0.025)
Year	N	Y	N	Y
City	N	Y	N	Y
N	187	187	187	187
adj. R2	0.751	0.910	0.667	0.713

Note: Standard errors in parentheses. * *p* < 0.1, ** *p* < 0.05, *** *p* < 0.01.

**Table 4:** The relationship between the economic development and environmental regulation (not the spatial result).

Var.	(11)	(12)	(13)	(14)	(15)	(16)	(17)	(18)	(19)	(20)	(21)	(22)
	W1				W2				W3			
	SAR	SEM	SDM	SIV	SAR	SEM	SDM	SIV	SAR	SEM	SDM	SIV
lnpgdp	3.388 ***	5.959 ***	3.910 ***	3.763 ***	3.578 ***	3.775 ***	4.165 ***	3.150 ***	3.648 ***	3.002 ***	5.525 ***	3.320 ***
	(4.81)	(9.47)	(5.20)	(0.690)	(4.97)	(5.20)	(4.77)	(0.716)	(4.96)	(4.34)	(5.96)	(0.646)
lnpgdpsq	−0.158 ***	−0.264 ***	−0.182 ***	−0.174 ***	−0.167 ***	−0.176 ***	−0.191 ***	−0.134 ***	−0.170 ***	−0.145 ***	−0.238 ***	−0.152 ***
	(−4.63)	(−8.82)	(−5.25)	(0.033)	(−4.80)	(−5.04)	(−4.69)	(0.034)	(−4.79)	(−4.26)	(−5.80)	(0.031)
lnpop	0.182	−0.450	−0.250	0.352	0.311	0.392	0.658	0.216	0.217	0.240	0.004	0.286
	(0.51)	(−1.47)	(−0.73)	(0.385)	(0.84)	(1.05)	(1.19)	(0.403)	(0.58)	(0.66)	(0.01)	(0.364)
lnmanu	0.188	0.004	−0.117	0.242 *	0.198	0.171	0.112	0.333 **	0.203	0.285 **	0.091	0.238 *
	(1.48)	(0.03)	(−1.22)	(0.134)	(1.51)	(1.30)	(1.06)	(0.139)	(1.51)	(2.16)	(0.76)	(0.128)
lnsevi	0.725 ***	0.203	−0.108	0.903 ***	0.787 ***	0.755 ***	0.689 *	1.138 ***	0.766 ***	0.886 ***	0.434	0.898 ***
	(2.80)	(0.89)	(−0.44)	(0.300)	(2.97)	(2.87)	(1.70)	(0.298)	(2.83)	(3.18)	(1.09)	(0.272)
lndens	0.457 ***	0.668 ***	0.411 ***	0.453 ***	0.454 ***	0.446 ***	0.310 **	0.396 ***	0.444 ***	0.480 ***	0.381 ***	0.431 ***
	(4.09)	(7.20)	(3.34)	(0.128)	(3.94)	(3.76)	(2.37)	(0.129)	(3.77)	(4.24)	(3.35)	(0.122)
lnstu	−0.407 ***	−0.487 ***	−0.302 ***	−0.390 ***	−0.409 ***	−0.423 ***	−0.420 ***	−0.368 ***	−0.418 ***	−0.352 ***	−0.376 ***	−0.394 ***
	(−7.97)	(−11.14)	(−5.44)	(0.060)	(−7.74)	(−7.88)	(−3.92)	(0.064)	(−7.77)	(−6.45)	(−4.41)	(0.057)
lnhealth	−0.003	0.017	0.027	0.002	−0.012	0.001	0.057	−0.015	−0.010	−0.041	0.054	−0.004
	(−0.07)	(0.48)	(0.60)	(0.047)	(−0.28)	(0.03)	(0.54)	(0.046)	(−0.24)	(−0.98)	(0.58)	(0.043)
lnbed	−0.051	−0.302 *	−0.320	0.018	−0.051	−0.053	0.053	0.193	−0.133	−0.155	−0.230	0.077
	(−0.27)	(−1.92)	(−1.64)	(0.181)	(−0.26)	(−0.27)	(0.22)	(0.187)	(−0.67)	(−0.85)	(−0.79)	(0.173)
lndoctor	0.119	0.019	−0.028	−0.003	0.129	0.118	0.059	0.010	0.151	0.165	−0.040	0.041
	(0.80)	(0.15)	(−0.19)	(0.168)	(0.84)	(0.75)	(0.38)	(0.168)	(0.96)	(1.19)	(−0.28)	(0.153)
lnconsp	−0.144 ***	−0.136 ***	−0.039 *	−0.149 ***	−0.147 ***	−0.154 ***	−0.167 ***	−0.126 ***	−0.152 ***	−0.108 ***	−0.179 ***	−0.137 ***
	(−6.64)	(−7.65)	(−1.73)	(0.026)	(−6.55)	(−6.86)	(−2.76)	(0.028)	(−6.65)	(−4.40)	(−3.23)	(0.024)
L.lnregu			0.511 ***				0.703 ***				0.723 ***	
			(1.10)				(1.68)				(1.11)	
Spatial rho	−0.512 **		0.751 ***	0.712 **	−0.185 **		−0.241 ***	0.904 **	−7.296 ***		−6.304 **	1.136 ***
	(−2.17)		(2.81)	(0.297)	(−1.02)		(−2.61)	(0.421)	(−4.32)		(−2.03)	(0.348)
lambda		−2.141 ***				−0.419 ***				−5.722 ***		
		(−1.87)				(−2.73)				(−3.80)		
Variancesigma2_e	0.018 ***	0.009 ***	0.006 ***	0.348 *	0.020 ***	0.019 ***	0.017 ***	−0.055 **	0.020 ***	0.024 ***	0.014 ***	0.186 **
	(9.50)	(8.73)	(10.40)	(0.203)	(9.63)	(9.51)	(6.71)	(0.191)	(10.18)	(8.24)	(5.01)	(0.231)
N	187	187	176	187	187	187	176	187	187	187	176	187
adj. R2	0.854	0.434	0.667	0.881	0.523	0.791	0.698	0.852	0.558	0.737	0.746	0.879

Note: In SAR, SEM, and SDM models, the t-values are in parentheses; in SIV models, standard errors are in parentheses; * *p* < 0.1, ** *p* < 0.05, *** *p* < 0.01.

**Table 5:** The technological innovation effect test.

Var.	(23)	(24)	(25)	(26)	(27)	(28)
	lnpatent	lnregu	lnpatent	lnregu	lnpatent	lnregu
	W1		W2		W3	
lnpgdp	0.855 ***		0.865 ***		0.668 ***	
	(0.132)		(0.119)		(0.124)	
lnpatent		1.056 ***		1.135 ***		1.132 ***
		(0.129)		(0.165)		(0.123)
lnpatentsq		−0.060 ***		−0.064 ***		−0.069 ***
		(0.008)		(0.010)		(0.008)
lnpop	0.071 **	0.086 ***	0.086 ***	0.085 ***	0.099 ***	0.098 ***
	(0.029)	(0.017)	(0.020)	(0.012)	(0.018)	(0.010)
lnmanu	−0.250	0.421 ***	−0.303 **	0.280 ***	−0.374 ***	0.354 ***
	(0.165)	(0.102)	(0.142)	(0.092)	(0.139)	(0.080)
lnsevi	−0.629	1.110 ***	−0.581	0.843 ***	−0.647 *	1.093 ***
	(0.409)	(0.221)	(0.385)	(0.222)	(0.376)	(0.194)
lndens	0.743 ***	0.216 ***	0.661 ***	0.278 ***	0.782 ***	0.302 ***
	(0.086)	(0.053)	(0.082)	(0.060)	(0.070)	(0.048)
lnstu	−0.297 ***	−0.359 ***	−0.226 ***	−0.287 ***	−0.270 ***	−0.383 ***
	(0.071)	(0.031)	(0.067)	(0.029)	(0.067)	(0.026)
lnhealth	0.039	0.056 ***	0.175 ***	0.114 ***	0.055	0.048 **
	(0.042)	(0.021)	(0.047)	(0.024)	(0.038)	(0.019)
lnbed	0.715 ***	0.260 ***	0.844 ***	0.627 ***	0.659 ***	0.379 ***
	(0.136)	(0.081)	(0.220)	(0.125)	(0.130)	(0.074)
lndoctor	−0.183	−0.277 ***	−0.360	−0.518 ***	−0.019	−0.296 ***
	(0.195)	(0.096)	(0.224)	(0.135)	(0.177)	(0.097)
lnconsp	−0.055 **	0.033 **	−0.068 **	0.014	−0.063 **	0.026 *
	(0.028)	(0.016)	(0.027)	(0.017)	(0.025)	(0.014)
Year	Y	Y	Y	Y	Y	Y
City	Y	Y	Y	Y	Y	Y
Spatial rho	1.130 ***	1.182 **	1.404 **	1.673 ***	3.012 ***	1.599 ***
	(0.377)	(0.495)	(0.593)	(0.355)	(0.483)	(0.558)
Variance sigma2_e	0.165 **	1.355 ***	0.250 ***	−1.043 ***	0.108 ***	−1.500 ***
	(0.028)	(0.456)	(0.411)	(0.241)	(0.036)	(0.270)
N	187	187	187	187	187	187
adj. R2	0.955	0.890	0.952	0.872	0.955	0.895

Note: Standard errors in parentheses, * *p* < 0.1, ** *p* < 0.05, *** *p* < 0.01.

**Table 6:** The resource consumption effect test.

Var.	(29)	(30)	(31)	(32)	(33)	(34)
	lnresource	lnregu	lnresource	lnregu	lnresource	lnregu
	W1		W2		W3	
lnpgdp	7.205 ***		8.610 ***		5.582 ***	
	(1.137)		(1.054)		(1.231)	
lnpgdpsq	−0.350 ***		−0.437 ***		−0.284 ***	
	(0.054)		(0.048)		(0.059)	
lnresource		0.299 ***		0.264 ***		0.307 ***
		(0.049)		(0.063)		(0.062)
lnpop	1.711 ***	−0.841 ***	0.303 ***	−0.235	1.971 ***	−0.618 *
	(0.498)	(0.268)	(0.024)	(0.359)	(0.573)	(0.342)
lnmanu	−0.110	0.503 ***	−0.081	0.567 ***	−0.228	0.568 ***
	(0.186)	(0.091)	(0.217)	(0.096)	(0.185)	(0.095)
lnsevi	−0.713	0.828 **	−1.981 ***	1.145 ***	−0.656 *	0.734 ***
	(0.504)	(0.332)	(0.404)	(0.256)	(0.383)	(0.261)
lndens	0.248 *	0.520 ***	0.771 ***	0.283 **	0.235	0.360 ***
	(0.143)	(0.093)	(0.085)	(0.136)	(0.166)	(0.123)
lnstu	−0.232 ***	−0.231 ***	−0.134 **	−0.264 ***	−0.211 ***	−0.215 ***
	(0.066)	(0.042)	(0.067)	(0.064)	(0.075)	(0.059)
lnhealth	0.010	0.058 *	0.058	−0.032	0.046	−0.019
	(0.053)	(0.035)	(0.051)	(0.044)	(0.060)	(0.045)
lnbed	0.242	0.032	−0.959 ***	0.130	0.424	0.105
	(0.246)	(0.148)	(0.219)	(0.199)	(0.283)	(0.201)
lndoctor	−0.079	−0.093	0.767 ***	0.094	0.010	−0.083
	(0.191)	(0.126)	(0.219)	(0.174)	(0.226)	(0.179)
lnconsp	−0.101 ***	−0.086 ***	−0.176 ***	−0.119 ***	−0.158 ***	−0.147 ***
	(0.028)	(0.019)	(0.027)	(0.026)	(0.031)	(0.025)
Year	Y	Y	Y	Y	Y	Y
City	Y	Y	Y	Y	Y	Y
Spatial rho	−5.762 ***	−5.451 ***	4.447 ***	−3.309 ***	−1.704 ***	9.719
	(0.644)	(0.499)	(0.557)	(1.038)	(0.437)	(7.061)
Variance sigma2_e	3.311 ***	1.201 ***	2.234 *	0.515 *	4.104 ***	0.790 ***
	(1.598)	(0.387)	(1.248)	(0.295)	(1.313)	(0.294)
N	187	187	187	187	187	187
adj. R2	0.986	0.865	0.945	0.919	0.981	0.917

Note: Standard errors in parentheses, * *p* < 0.1, ** *p* < 0.05, *** *p* < 0.01.

**Table 7:** The effect decomposition.

Var.	(35)	(36)	(37)	(38)	(39)	(40)
	lnregu	lnregu	lnregu	lnregu	lnregu	lnregu
	W1		W2		W3	
lnpgdp	0.234	2.273 **	0.036	0.763	0.211	0.932
	(0.878)	(0.923)	(0.818)	(0.936)	(0.842)	(0.845)
lnpgdpsq	−0.022	−0.084 **	−0.014	−0.048	−0.021	−0.046
	(0.040)	(0.041)	(0.037)	(0.043)	(0.038)	(0.039)
lnpatent	0.937 ***	0.495 ***	1.163 ***	1.476 ***	0.962 ***	1.324 ***
	(0.180)	(0.182)	(0.171)	(0.236)	(0.176)	(0.175)
lnpatentsq	−0.050 ***	−0.027 ***	−0.063 ***	−0.078 ***	−0.051 ***	−0.072 ***
	(0.010)	(0.010)	(0.010)	(0.013)	(0.010)	(0.010)
lnresource	0.175 ***	0.204 ***	0.130 **	0.124 **	0.142 ***	0.133 ***
	(0.053)	(0.048)	(0.053)	(0.056)	(0.054)	(0.050)
lnpop	−0.318	−1.025 ***	−0.302	0.185	−0.257	−0.448
	(0.353)	(0.340)	(0.349)	(0.403)	(0.345)	(0.343)
lnmanu	0.257 **	0.160	0.255 **	0.105	0.261 **	0.127
	(0.116)	(0.112)	(0.115)	(0.134)	(0.114)	(0.119)
lnsevi	0.790 ***	0.324	0.817 ***	0.512 *	0.828 ***	0.304
	(0.254)	(0.235)	(0.250)	(0.278)	(0.246)	(0.250)
lndens	0.590 ***	0.558 ***	0.580 ***	0.494 ***	0.583 ***	0.570 ***
	(0.118)	(0.106)	(0.117)	(0.140)	(0.117)	(0.112)
lnstu	−0.313 ***	−0.316 ***	−0.330 ***	−0.270 ***	−0.336 ***	−0.296 ***
	(0.056)	(0.049)	(0.056)	(0.061)	(0.054)	(0.051)
lnhealth	−0.016	−0.002	−0.039	−0.036	−0.037	−0.005
	(0.041)	(0.037)	(0.039)	(0.042)	(0.039)	(0.038)
lnbed	0.044	−0.054	0.042	−0.110	0.082	−0.152
	(0.156)	(0.167)	(0.152)	(0.201)	(0.154)	(0.181)
lndoctor	0.088	−0.032	0.198	0.238	0.175	0.085
	(0.146)	(0.135)	(0.143)	(0.155)	(0.141)	(0.142)
lnconsp	−0.095 ***	−0.103 ***	−0.100 ***	−0.113 ***	−0.093 ***	−0.104 ***
	(0.025)	(0.022)	(0.025)	(0.026)	(0.024)	(0.022)
Year		Y		Y		Y
City		Y		Y		Y
Spatial rho	0.191	−0.727	0.767 **	0.479	0.751 **	−5.313
	(0.245)	(0.443)	(0.338)	(0.703)	(0.328)	(3.703)
Variance sigma2_e	0.378 **	4.845 ***	0.233	1.009 ***	0.189	−9.257 ***
	(0.148)	(0.825)	(0.146)	(0.365)	(0.181)	(1.591)
N	187	187	187	187	187	187

Note: Standard errors in parentheses, * *p* < 0.1, ** *p* < 0.05, *** *p* < 0.01.

**Table 8:** The summary of effects.

Effect Decomposition	W1	W2	W3
Total effect	3.589	3.016	3.168
Innovation effect	0.996	1.071	1.063
Resource effect	0.299	0.264	0.307
Direct effect	2.189	0.000	0.000
Indirect effect	0.527	1.530	1.372

## Data Availability

The data presented in this study are openly available in China’s City Statistics Yearbook, and information collected by crawler technology.
